# Association of the insulin resistance marker triglyceride glucose index with migraine: results of a cross-sectional and prospective cohort study

**DOI:** 10.22514/jofph.2025.017

**Published:** 2025-03-12

**Authors:** Jielong Wu, Xiaodong Yuan, Jiedong Zhao, Yeting Wu, Dan Chen, Lingshan Ma, Chuya Jing, Liangcheng Zheng, Xingkai An, Qing Lin, Zhanxiang Wang, Qilin Ma, Jie Fang

**Affiliations:** ^1^Department of Neurology and Department of Neuroscience, The First Affiliated Hospital of Xiamen University, School of Medicine, Xiamen University, 361003 Xiamen, Fujian, China; ^2^National Institute for Data Science in Health and Medicine, Xiamen University, 361102 Xiamen, Fujian, China; ^3^Department of Gynecology of Xiamen Maternal and Child Health Care Hospital, 361003 Xiamen, Fujian, China; ^4^The Graduate School of Fujian Medical University, 350122 Fuzhou, Fujian, China; ^5^The School of Clinical Medicine, Fujian Medical University, 350108 Fuzhou, Fujian, China; ^6^Fujian Key Laboratory of Brain Tumors Diagnosis and Precision Treatment, 361003 Xiamen, Fujian, China; ^7^Xiamen Key Laboratory of Brain Center, 361003 Xiamen, Fujian, China; ^8^Xiamen Medical Quality Control Center for Neurology, 361003 Xiamen, Fujian, China; ^9^Fujian Provincial Clinical Research Center for Brain Diseases, 361003 Xiamen, Fujian, China; ^10^Xiamen Clinical Research Center for Neurological Diseases, 361003 Xiamen, Fujian, China; ^11^Department of Neurosurgery and Department of Neuroscience, The First Affiliated Hospital of Xiamen University, School of Medicine, Xiamen University, 361003 Xiamen, Fujian, China

**Keywords:** Chinese population, Migraine incidence, Migraine disability, Triglyceride glucose index, Insulin resistance

## Abstract

**Background**: Type 2 diabetes has been shown to reduce the risk of migraine, whereas insulin resistance (IR) is often elevated in migraineurs. The triglyceride glucose index (TyG) serves as a reliable surrogate marker of IR, which has been hypothesized to be associated with migraine pathophysiology. This study aimed to examine the relationship between TyG index scores and incidence as well as the severity of migraines through both cohort and cross-sectional analyses. **Methods**: Using data from the China Health and Retirement Longitudinal Study (CHARLS), we evaluated migraine incidence between 2015 and 2020. TyG index values were calculated using the following formula: ln[fasting triglyceride (mg/dL) × fasting glucose (mg/dL)/2]. The impact of TyG index scores on the incidence of migraines was assessed using a multivariate-adjusted Cox regression model. The cross-sectional study included 161 patients with migraines in Xiamen, China. The relationship between TyG index scores and different migraine characteristics was examined using multivariable and ordered logistic regression models with further subgroup analysis by migraine course. **Results**: Among the cohort participants, 1001 new migraine cases were identified during follow-up, with no significant relationship found between TyG index scores and migraine incidence (hazard ratio = 1.024 (0.916, 1.145), *p* = 0.677 > 0.05). The cross-sectional study showed that migraine-related disability was significantly lower among patients in the second, third and fourth quartiles of TyG index scores compared to the first quartile (odds ratio = 0.402 (0.163, 0.973), 0.322 (0.128, 0.789) and 0.301 (0.119, 0.736), respectively; *p*_trend_ = 0.009). A similar trend was observed in patients with migraine history of less than ten years. **Conclusions**: Integrating the results of both cohort and cross-sectional studies, this study suggests that IR may play a protective role in the early stages of migraine. Further research into the relevant underlying mechanisms could aid in identifying new therapeutic targets for migraine management.

## 1. Introduction

Migraine is a recurrent and highly prevalent disabling primary headache, which can lead to severe disability and impose a huge burden on affected individuals [1, 2]. The pathophysiology of migraine involves a complex interplay of genetic factors and activation of trigeminovascular system, resulting in nervous system sensitization and abnormal neuronal responses. Multiple factors, including genetic, hormonal, and environmental contributors are implicated in its onset and progression. As the pathophysiology of migraine remains incompletely understood [3], and a substantial proportion of patients fail to achieve satisfactory outcomes with existing treatments [4], novel therapeutic targets and alternative treatment strategies are urgently needed.

Type 2 diabetes is a heterogeneous condition marked by high blood sugar levels and insulin resistance (IR). Epidemiological and genetic evidence suggests an association between type 2 diabetes and migraine incidence. In cohort studies conducted in France and Norway, the incidence of migraine was found to be significantly lower in patients with type 2 diabetes than in controls, with consistent results when stratified by sex [5, 6, 7]. The results of a two-way Mendelian randomization study based on the latest migraine genome-wide association study (GWAS) data suggested a causal association between type 2 diabetes and IR and a lower risk of migraine [8, 9]. Conversely, numerous observational studies have indicated that individuals with migraines exhibit a higher proportion of IR compared to healthy control groups [10, 11, 12, 13, 14, 15, 16]. These studies also suggest that treatment for IR may help to relieve migraine symptoms. Thus, the apparent paradox of type 2 diabetes reducing migraine incidence while IR is elevated IR in migraineurs is worthy of attention. IR plays a key role in migraine pathophysiology, linking migraines with comorbidities such as obesity and depression [17]. By disrupting brain glucose metabolism, IR creates energy deficits and neuronal stress, triggering inflammation that further worsens IR. This “neuroenergetic” dysfunction may bridge episodic and chronic migraines [18], suggesting new directions for targeted treatment development.

The triglyceride glucose index (TyG), which uses fasting glucose and triglyceride levels, offers a practical and accessible alternative to homeostasis model assessment of insulin resistance (HOMA-IR) for assessing insulin resistance [19, 20]. Studies have reported its utility in diagnosing and predicting new-onset diabetes [21, 22], and a growing number of studies have reported a strong link between TyG index scores and conditions such as cardiovascular disease [23], stroke [24], depression [24] and dementia [25]. However, to the best of our knowledge, no studies have examined the relationship between TyG index scores and migraine incidence in the Chinese population, and there remains a significant lack of research on TyG index levels in patients with migraines.

Therefore, this study aimed to fill this gap by evaluating the association between TyG index scores and migraine risk in a cohort study and examining the relationship between TyG index scores and migraine severity in a cross-sectional study. We hypothesize that a higher TyG index is linked with a lower risk of migraines and a lower degree of disability in patients with migraine. By elucidating the role of IR using TyG index scores, this study seeks to provide novel insights into migraine pathophysiology and potentially contribute to the development of future therapeutic strategies.

## 2. Methods

### 2.1 Study design and participants

The cohort study utilized follow-up data from the China Health and Retirement Longitudinal Study (CHARLS) [26]. The fifth wave of CHARLS data, collected in 2020, was officially released on 16 November 2023, providing extensive, high-quality microdata on Chinese households and individuals 45 years of age and older. In this study, 2015 was designated as the baseline. We retrospectively compiled essential demographic characteristics, medical histories, and blood analysis data on the participants. Migraine incidence was assessed using follow-up data from 2018 and 2020. The exclusion criteria in the cohort study were (1) participants who missed any surveys between 2011 and 2020, (2) those lacking biomarker data for fasting glucose and triglyceride levels in the 2011 (wave 1) and 2015 (wave 3) sessions, (3) individuals missing age data, and (4) those who already had migraines at the study’s start. Ultimately, the cohort study consisted of 8523 individuals. A flowchart detailing the participant selection process for the cohort study is presented in Fig. 1.

**Fig. 1.** Flowchart of the cohort study. TyG: triglyceride glucose index; TG: triglyceride; FBG: fasting blood glucose.

As part of the research efforts, a cross-sectional study was undertaken in Xiamen, China. This study included 161 patients with migraines who attended the Headache Clinic at the First Affiliated Hospital of Xiamen University from April 2021 to December 2022. Neurologists diagnosed migraines based on the International Classification of Headache Disorders-3 (ICHD-3) guidelines, further classifying cases by the presence or absence of aura [27]. The exclusion criteria in the cross-sectional analysis were (1) individuals with chronic migraines and (2) those who self-identified as having type 2 diabetes, as diabetes could confound TyG index analysis. The participants underwent routine blood biochemical testing during their initial visit and were interviewed face-to-face using a structured questionnaire.

### 2.2 Assessment of the TyG index

The key factor of concern was IR, which was evaluated using the TyG index. In the cohort study, TyG index scores were meticulously determined using blood data obtained in 2015. We strategically supplemented this data with blood data from 2011 to address any missing data and ensure a robust and comprehensive analysis. In the cross-sectional study, all study subjects had fasting blood glucose and fasting triglyceride assessments at the initial clinic visit. TyG index scores were determined using the formula: ln[fasting triglyceride (mg/dL) × fasting glucose (mg/dL)/2] [28]. Additionally, the participants were categorized into four distinct groups based on TyG index level quartiles.

### 2.3 Assessment of migraine incidence

In the cohort study, all individuals underwent follow-up surveys in both 2018 and 2020, and migraine incidence was evaluated from 2015 to 2020. Consistent with our previous research, individuals who self-reported “yes” to the questions “currently feeling stomach pain” and “currently feeling head pain” were classified as having migraines. The aforementioned items in CHARLS align with the diagnostic criteria for migraines in the ID Migraine questionnaire, both exhibiting high sensitivity and specificity [29].

### 2.4 Assessment of migraine severity

In the cross-sectional study, migraine severity was assessed across four dimensions: duration, frequency, pain intensity and disability. The participants were asked to describe their typical headache characteristics, including average migraine duration (in hours), frequency (days per month), pain intensity and the level of disability caused by the migraines. Consistent with previous research, migraine duration was categorized as ≤10 hours (reference group) and >10 hours. Frequency was classified as an ordered categorical variable of <4 days per month (reference group), 4–8 days per month and >8 days per month. Pain intensity was measured using visual analog scale (VAS) scores and divided into mild or moderate (VAS <8, reference group) and severe (VAS ≥8). Migraine disability was assessed using the Migraine Disability Assessment Questionnaire (MIDAS), which evaluates the impact of migraines on patients over the preceding 3 months [30] and was divided into three levels as an ordered categorical variable as follows: mild (MIDAS ≤10, reference group), moderate (10 < MIDAS ≤ 20) and severe (MIDAS >20).

### 2.5 Covariates

The cohort study considered several covariates: (1) sociodemographic factors, such as age, gender (female or male), education level (illiterate, primary or higher), residence (urban or rural) and marital status (married or unmarried); (2) health-related factors, including self-reported health status (good or poor), alcohol consumption (yes or no), smoking habits (yes or no), participation in social activities (yes or no), exercise habits (yes or no), the presence of depression (yes or no) and sleep duration (≤6 hours per day, 6–8 hours per day, or >8 hours per day); and (3) disease conditions and medications including obesity, diabetes, hypertension, dyslipidemia, heart disease, stroke, kidney disease, liver disease, diabetes medications, hypertension medications and dyslipidemia medications, which were dichotomized as yes and none. Older adults were defined as age ≥60 years. Depression was evaluated using the 10-item Center for Epidemiological Studies Depression Scale (CESD-10). Obesity was determined according to the World Health Organization’s recommendation for Asian and South Asian populations (body mass index (BMI) ≥25 kg/m^2^). Diabetes was identified through biomarker data, with diagnostic criteria including fasting blood glucose levels of ≥126 mg/dL or a hemoglobin A1c (HbA1c) of ≥6.5% or above. Conditions such as hypertension, dyslipidemia, heart disease, stroke, kidney disease and liver disease were recorded if the participants reported a prior medical diagnosis. Medication usage, including treatments for diabetes, hypertension and hyperlipidemia, was self-reported by the participants.

The cross-sectional study incorporated various covariates: sex, age, educational attainment (categorized as primary, secondary or tertiary), BMI, self-reported family economic status (low, middle or high-income), self-assessed life satisfaction (poor, fair or well), the duration of migraine history (in years), familial migraine history, aura presence and medication patterns. Migraine with aura was defined based on the ICHD-3 criteria, characterised by the occurrence of specific aura symptoms before the onset of headache. Medication usage encompassed both acute treatments (such as the use of pain relievers during headache episodes) and preventive medications administrated during headache-free intervals.

### 2.6 Statistical analysis

Participant characteristics in both studies were stratified into TyG index quartiles. Statistical analyses employed Pearson’s chi-squared test, Fisher’s exact test, and one-way analysis of variance (ANOVA) to evaluate differences across TyG index quartiles. In the cross-sectional component, we created box and violin plots using ggstatsplot in R package to visualize the relationship between TyG index levels and various headache characteristics (including duration, frequency, pain intensity and associated disability). Given the non-normal distribution of data, nonparametric methods were applied, with TyG index values presented as median and interquartile range.

In the cohort study, Cox proportional hazards models were employed to evaluate the association between TyG index scores and migraine incidence, yielding hazard ratios (HR) with 95% confidence intervals (CIs). The results were visualized using forest plots generated using forestplot in R package. All analyses were adjusted for previously mentioned covariates. Multivariable and ordered logistic regression models were used to examine the relationship between TyG index scores and migraine characteristics in the cross-sectional study and calculate odds ratios (ORs) with 95% CIs. The analysis comprised two models: model 1 was an unadjusted analysis examining TyG index score effects on migraine duration, frequency, pain intensity and disability, while model 2 was a fully adjusted analysis incorporating all of the aforementioned covariates. TyG index scores were analyzed both as continuous variables and as quartile-based categories (Q1–Q4), with Q1 serving as the reference group. Trend analysis was conducted by treating quartiles as ordinal values (1–4). Additional regression analysis was stratified by migraine course. All statistical analyses were performed using R version 4.3.2 (2023 R Foundation for Statistical Computing), with statistical significance set at *p* < 0.05.

## 3. Results

### 3.1 Characteristics of individuals in the cohort study

Of the 8523 CHARLS participants included in the cohort study, 53.77% were female and 56.31% were older adults, with a mean (standard deviation (SD)) age of 61.36 (8.87) years. The mean TyG index score was 8.70 ± 0.63. As shown in Table 1, higher TyG index scores were significantly linked to being female, having a higher educational level, residing in urban areas, being married, having no alcohol consumption or smoking, engaging in more social activities, exercising less, experiencing depression, having longer sleep durations, conditions such as obesity, diabetes, dyslipidemia, hypertension, heart disease, stroke, kidney disease and not receiving medications or treatments for diabetes or dyslipidemia.

**Table 1.** Characteristics of individuals according to the quartiles of TyG index.

Characteristic		Overall, N = 8523^1^	Q1, N = 2131^1^	Q2, N = 2129^1^	Q3, N = 2134^1^	Q4, N = 2129^1^	*p*-value^2^
TyG index		8.70 ± 0.63	7.98 ± 0.22	8.43 ± 0.10	8.83 ± 0.13	9.56 ± 0.43	<0.001
Older adults		4799 (56.31%)	1231 (57.77%)	1227 (57.63%)	1182 (55.39%)	1159 (54.44%)	0.068
Female		4583 (53.77%)	934 (43.83%)	1137 (53.41%)	1253 (58.72%)	1259 (59.14%)	<0.001
Illiterate or primary		7637 (89.60%)	1933 (90.71%)	1918 (90.09%)	1897 (88.89%)	1889 (88.73%)	0.100
Urban Residence		2913 (34.18%)	575 (26.98%)	692 (32.50%)	744 (34.86%)	902 (42.37%)	<0.001
Married		7084 (83.12%)	1758 (82.50%)	1733 (81.40%)	1789 (83.83%)	1804 (84.73%)	0.020
Good health status		1974 (23.16%)	520 (24.40%)	502 (23.58%)	501 (23.48%)	451 (21.18%)	0.077
Drinking		3852 (45.20%)	1074 (50.40%)	985 (46.27%)	911 (42.69%)	882 (41.43%)	<0.001
Smoking		3690 (43.29%)	1093 (51.29%)	919 (43.17%)	832 (38.99%)	846 (39.74%)	<0.001
Social activity		3996 (46.88%)	918 (43.08%)	991 (46.55%)	1012 (47.42%)	1075 (50.49%)	<0.001
Exercise		1518 (17.81%)	458 (21.49%)	416 (19.54%)	342 (16.03%)	302 (14.19%)	<0.001
Depressive symptom		2560 (30.04%)	676 (31.72%)	637 (29.92%)	603 (28.26%)	644 (30.25%)	0.104
Sleep duration (h)							
	≤6	2924 (34.31%)	727 (34.12%)	785 (36.87%)	745 (34.91%)	667 (31.33%)	0.011
	6–8	3367 (39.50%)	833 (39.09%)	828 (38.89%)	830 (38.89%)	876 (41.15%)	
	>8	2232 (26.19%)	571 (26.79%)	516 (24.24%)	559 (26.19%)	586 (27.52%)	
Obesity		2746 (32.22%)	340 (15.95%)	553 (25.97%)	814 (38.14%)	1039 (48.80%)	<0.001
Diabetes		1508 (17.69%)	141 (6.62%)	203 (9.53%)	364 (17.06%)	800 (37.58%)	<0.001
Dyslipidemia		1622 (19.03%)	210 (9.85%)	308 (14.47%)	472 (22.12%)	632 (29.69%)	<0.001
Hypertension		2821 (33.10%)	506 (23.74%)	620 (29.12%)	769 (36.04%)	926 (43.49%)	<0.001
Heart disease		1405 (16.48%)	285 (13.37%)	324 (15.22%)	377 (17.67%)	419 (19.68%)	<0.001
Stroke		289 (3.39%)	49 (2.30%)	56 (2.63%)	83 (3.89%)	101 (4.74%)	<0.001
Kidney disease		742 (8.71%)	208 (9.76%)	203 (9.53%)	148 (6.94%)	183 (8.60%)	0.004
Liver disease		501 (5.88%)	143 (6.71%)	107 (5.03%)	115 (5.39%)	136 (6.39%)	0.061
Hypertension medications		7683 (90.14%)	1944 (91.22%)	1933 (90.79%)	1903 (89.18%)	1903 (89.38%)	0.059
Diabetes medications		7423 (87.09%)	2001 (93.90%)	1954 (91.78%)	1858 (87.07%)	1610 (75.62%)	<0.001
Dyslipidemia medications		7559 (88.69%)	1990 (93.38%)	1924 (90.37%)	1856 (86.97%)	1789 (84.03%)	<0.001

*^1^Mean ± SD; n (%); ^2^One-way ANOVA; Fisher’s exact test; Pearson’s Chi-squared test; Q1/Q2/Q3/Q4 represents the quartile of TyG index. TyG: triglyceride glucose index.*

### 3.2 Impact of TyG index scores on migraine incidence

During the cohort study follow-up period, 1001 new migraine cases were identified. Fig. 2 illustrates the influence of TyG index scores on migraine risk. After adjusting for the previously mentioned covariates, no significant association was observed between TyG index scores and the incidence of migraines (in model 3, HR = 1.024 (0.916, 1.145), *p *= 0.677 > 0.05).

**Fig. 2.** The effect of TyG index on migraine incidence. Results was assessed using the Cox proportional hazards model. HR: hazard ratio; CI: confidence interval; TyG: triglyceride glucose index.

### 3.3 Characteristics of patients with migraines in the cross-sectional study

In the cross-sectional study, 88.20% (142 out of 161) of the patients with migraines were female, with an average age of 34.74 years (SD = 10.06). The average TyG index score was 8.33 (±0.60), and the average duration of migraine history was 10.83 years (±9.01). In the examination of TyG index quartiles, no significant differences were observed in terms of sex distribution, educational level, family economic status, life satisfaction, migraine course, family history of migraine, the presence or absence of aura or medication use. However, higher age and BMI were significantly associated with higher TyG index scores (*p* < 0.05) (Table 2).

**Table 2.** Characteristics of migraine patients according to the quartiles of TyG index.

Characteristic		Overall, N = 161^1^	Q1, N = 41^1^	Q2, N = 40^1^	Q3, N = 39^1^	Q4, N = 41^1^	*p*-value^2^
TyG index		8.33 ± 0.60	7.66 ± 0.18	8.08 ± 0.11	8.43 ± 0.12	9.14 ± 0.40	<0.001
Female		142 (88.20%)	37 (90.24%)	36 (90.00%)	37 (94.87%)	32 (78.05%)	0.147
Age (yr)		34.74 ± 10.06	30.90 ± 8.06	33.98 ± 9.06	38.90 ± 11.69	35.37 ± 9.86	0.004
BMI (kg/m^2^)		22.11 ± 3.10	21.14 ± 2.57	22.07 ± 2.48	21.65 ± 2.81	23.57 ± 3.86	0.002
Educational level							
	Primary	15 (9.32%)	0 (0.00%)	4 (10.00%)	6 (15.38%)	5 (12.20%)	0.228
	Secondary	48 (29.81%)	14 (34.15%)	11 (27.50%)	12 (30.77%)	11 (26.83%)	
	Tertiary	98 (60.87%)	27 (65.85%)	25 (62.50%)	21 (53.85%)	25 (60.98%)	
Family economic status							
	Low-income	12 (7.45%)	1 (2.44%)	6 (15.00%)	2 (5.13%)	3 (7.32%)	0.082
	Middle-income	137 (85.09%)	38 (92.68%)	33 (82.50%)	35 (89.74%)	31 (75.61%)	
	High-income	12 (7.45%)	2 (4.88%)	1 (2.50%)	2 (5.13%)	7 (17.07%)	
Life satisfaction							
	Poor	62 (38.51%)	18 (43.90%)	9 (22.50%)	19 (48.72%)	16 (39.02%)	0.182
	Fair	69 (42.86%)	18 (43.90%)	19 (47.50%)	15 (38.46%)	17 (41.46%)	
	Well	30 (18.63%)	5 (12.20%)	12 (30.00%)	5 (12.82%)	8 (19.51%)	
Migraine course (yr)		10.83 ± 9.01	8.29 ± 6.60	11.35 ± 10.26	12.46 ± 9.31	11.32 ± 9.30	0.189
Family history of migraine		66 (40.99%)	16 (39.02%)	20 (50.00%)	15 (38.46%)	15 (36.59%)	0.606
Migraine with aura		16 (9.94%)	5 (12.20%)	2 (5.00%)	3 (7.69%)	6 (14.63%)	0.484
Medication use		41 (25.47%)	7 (17.07%)	11 (27.50%)	11 (28.21%)	12 (29.27%)	0.557

*^1^Mean ± SD; n (%); ^2^One-way ANOVA; Fisher’s exact test; Pearson’s Chi-squared test; Q1/Q2/Q3/Q4 represents the quartile of TyG index. TyG: triglyceride glucose index; BMI: body mass index.*

### 3.4 TyG index and migraine characteristics

We analyzed the relationship between TyG index levels and various migraine characteristics, including attack duration, frequency, pain severity and associated disability (Fig. 3). While variations in these parameters were observed, statistical analysis revealed no meaningful correlation between TyG index scores and either migraine frequency or pain intensity levels. However, patients with shorter migraine durations (≤10 hours) reported the highest TyG index levels compared to those with longer durations (8.452 (8.044, 8.832) *vs.* 8.175 (7.823, 8.583), *p* = 0.019). Additionally, patients with mild migraine disability had higher TyG index levels than those with moderate or severe disabilities (8.355 (8.068, 8.739) *vs.* 8.092 (7.771, 8.656) *vs.* 8.148 (7.791, 8.474), *p* = 0.009).

**Fig. 3.** TyG index levels by different headache characteristics. (a) migraine duration. (b) migraine frequency (days/month). (c) pain intensity. (d) migraine disability. Nonparametric statistical methods were used, and the median and interquartile range of the TyG index were reported. TyG: triglyceride glucose index.

### 3.5 Logistic regression and subgroup analysis

The analysis of the influence of TyG index scores on migraine characteristics is presented in Table 3. Multivariable logistic regression, with adjustments for confounders, revealed no significant associations between TyG index scores and migraine duration, frequency or pain intensity. Notably, TyG index scores demonstrated a consistent and significant protective effect against migraine disability, both in unadjusted (OR = 0.559; 95% CI: 0.337–0.904) and adjusted analyses (OR = 0.510; 95% CI: 0.289–0.877). Additionally, patients in the second, third and fourth quartiles of the TyG index exhibited lower odds of experiencing migraine disability compared to those in the first quartile (OR = 0.402 (0.163, 0.973), 0.322 (0.128, 0.789) and 0.301 (0.119, 0.736), respectively; *p*_trend_ = 0.009).

**Table 3.** The association between TyG index and migraine characteristics*.

Variables		Model 1				Model 2			
		OR	95% CI	*p*	*p_trend_*	OR	95% CI	*p*	*p_trend_*
Migraine duration									
	TyG index	0.546	0.307, 0.954	0.035		0.728	0.372, 1.428	0.350	
	Q1	—	—		0.028	—	—		0.289
	Q2	0.639	0.220, 1.793	0.398		0.972	0.301, 3.121	0.962	
	Q3	0.485	0.169, 1.324	0.164		0.784	0.244, 2.473	0.678	
	Q4	0.342	0.122, 0.900	0.034		0.571	0.177, 1.764	0.335	
Migraine frequency									
	TyG index	0.812	0.435, 1.463	0.493		0.988	0.484, 1.953	0.971	
	Q1	—	—		0.364	—	—		0.798
	Q2	1.265	0.502, 3.248	0.626		1.108	0.398, 3.117	0.848	
	Q3	1.063	0.388, 2.893	0.901		1.124	0.368, 3.408	0.833	
	Q4	0.643	0.223, 1.788	0.401		0.824	0.242, 2.741	0.757	
Pain intensity									
	TyG index	0.839	0.492, 1.412	0.510		0.821	0.446, 1.490	0.517	
	Q1	—	—		0.194	—	—		0.166
	Q2	1.283	0.536, 3.097	0.576		1.373	0.519, 3.669	0.523	
	Q3	0.656	0.266, 1.591	0.353		0.537	0.196, 1.434	0.219	
	Q4	0.672	0.277, 1.610	0.374		0.650	0.236, 1.763	0.399	
Migraine disability									
	TyG index	0.559	0.337, 0.904	0.022		0.510	0.289, 0.877	0.019	
	Q1	—	—		0.007	—	—	—	0.009
	Q2	0.452	0.200, 1.006	0.056		0.402	0.163, 0.973	0.045	
	Q3	0.301	0.128, 0.688	0.005		0.322	0.128, 0.789	0.013	
	Q4	0.351	0.156, 0.776	0.012		0.301	0.119, 0.736	0.011	

*Model 1 analysis was non-adjusted; Model 2 analysis was adjusted for sex, age, educational level, BMI, family economic status, life satisfaction, migraine course, family history of migraine, presence or absence of aura and medication use. OR: Odds Ratio; CI: Confidence Interval; p_trend_: p for trend; Q1/Q2/Q3/Q4 represents the quartile of TyG index; TyG: triglyceride glucose index.

Fig. 4 presents a stratified analysis examining the influence of TyG index scores on migraine disability based on disease duration. For patients with fewer than 10 years of migraine history, the findings mirrored those of the overall study population, showing a significant inverse relationship between TyG index values and disability risk (model 1: *p*_trend_ = 0.014; model 2: *p*_trend_ = 0.009). In contrast, among patients with chronic migraines (≥10 years), TyG index values were not significantly associated with disability, regardless of confounding factor adjustments or whether the index values were analyzed as continuous or categorical variables.

**Fig. 4.** Subgroup analysis by migraine course. (a) migraine course <10 years. (b) migraine course ≥10 years. The symbols indicate point estimates of ORs and the vertical bars indicate the corresponding 95% CIs. Migraine patients in the TyG index 1st quartile being the reference category. The OR trends were evaluated by regarding the quartiles of TyG index levels as a continuous variable. Model 1 analysis was non-adjusted; Model 2 analysis was adjusted for sex, age, educational level, BMI, family economic status, life satisfaction, migraine course, family history of migraine, presence or absence of aura, and medication use. OR: Odds Ratio; TyG: triglyceride glucose index; CI: Confidence Interval.

## 4. Discussion

Our research comprised two distinct components: a prospective cohort study and a cross-sectional analysis. The prospective study followed 8523 Chinese individuals over 5 years, with assessments conducted at the third and fifth years, to investigate the relationship between baseline TyG index values and migraine development. This investigation did not support our initial hypothesis that elevated TyG index levels would reduce migraine risk. The cross-sectional component examined 161 patients with episodic migraines and analyzed the relationship between TyG index values and headache characteristics. We found that higher TyG index levels correlated with reduced migraine disability, particularly among patients who had experienced migraines for fewer than 10 years. This finding suggests that IR might play a protective role during the early stages of migraine development, potentially reducing the overall disease burden.

Previous cohort studies indicated that individuals with type 2 diabetes have a significantly reduced risk of developing migraines [5, 6, 7]. Given the well-established positive correlation between elevated TyG index levels and type 2 diabetes risk, we hypothesized that individuals with higher TyG index values would exhibit a significantly lower likelihood of developing migraines. However, our study results contradicted this hypothesis despite controlling for basic demographic characteristics, health-related factors, comorbidities and related medications. An analysis utilizing data from the National Health and Nutrition Examination Survey (NHANES) also investigated the relationship between TyG index levels and migraines [31]. Only after further adjustment for a range of migraine treatment medications did this study identify a protective effect of the TyG index score on migraines. Although this study included a smaller number of migraine cases and positive results were only observed in women and Mexican-Americans, it suggests the need to further examine the effect of migraine treatment medications on the outcomes. A cross-sectional study in Turkey, which included 150 migraine patients, found that metoclopramide treatment and the use of nonsteroidal anti-inflammatory drugs during an attack were significantly associated with IR [32]. Based on this background, we infer that factors such as migraine medications may lead to IR, which is reflected in a corresponding increase in the TyG index, potentially masking the true effect of TyG index scores on migraine incidence.

We conducted an additional cross-sectional study to further investigate the association between TyG index scores and the characteristics of migraines in patients without comorbid type 2 diabetes. In the current study, we used TyG index scores as a surrogate marker for IR. Among the migraine patients, we found a significant correlation between age, BMI, and TyG index scores. This is consistent with findings in other diseases, including cardiovascular diseases and diabetes [33, 34, 35, 36]. After adjusting for various confounding factors, such as migraine course, family history, aura and migraine medications, no significant correlation was found between the TyG index and migraine duration, migraine frequency and pain intensity in the regression analysis. One possible reason for these findings is the relatively small sample size of the study, which may have lacked sufficient power to detect intergroup differences. Nonetheless, the results suggest a significant association between TyG index scores and migraine disability, which is evaluated using a comprehensive scale of migraine disease burden.

Previous studies indicated a link between IR and migraines, with migraine patients having higher IR levels compared to healthy controls [10, 11, 12, 13, 14, 15, 16]. This suggests that IR may play a role in the pathogenesis of migraines, although the underlying mechanisms remain unclear. A study conducted in Egypt reported that the severity and impact of migraine attacks were heightened in patients with IR [16]. In our study, after adjusting for variables such as sex, age, educational level, BMI, family economic status, life satisfaction, duration of migraine, familial history of migraine, the presence or absence of aura, and medication usage we discovered that IR could mitigate the risk of migraine-related disability, particularly during the early stages of the disease. The opposing conclusions drawn from previous research maybe attributed to smaller sample sizes and inadequate adjustments for confounding variables in those investigations. Growing evidence indicates that migraines are caused by an imbalance between brain energy reserves and workload [18, 37, 38, 39]. In this context, IR or reduced insulin sensitivity may function as a temporary adaptive mechanism to maintain adequate brain energy supply [40, 41]. The combined findings of the current research and evidence from previous cohort studies support the hypothesis that IR is not a risk factor for migraines but rather a protective factor that helps compensate for brain energy demands after migraine attacks through adaptive responses.

The current study suggests the potential impact of the migraine course on the association between TyG index scores and migraine disability. The effect of TyG index scores on migraine disability was non-significant in patients with longer migraine courses. Previous research reported a significant correlation between longer migraine courses and increased disease burden, including an increased risk of impaired cardiac diastolic function, interictal osmophobia and migraine-related disability [42, 43, 44]. Furthermore, our recent study observed that younger patients with longer migraine durations (≥10 years) have higher levels of serum neurofilament light chain, suggesting possible nerve damage in this subgroup of patients [45]. Therefore, we speculate that the TyG index may have a positive impact in the early stages of migraine. However, as the disease progresses, patients may experience nerve damage and other associated symptoms, leading to a diminished significance of TyG index scores on migraine disability.

Several mechanisms may underlie the relationship between IR and migraines. Genetic predispositions could affect insulin receptor function, increasing susceptibility to IR patients with migraines [46]. Neuropeptides in the hypothalamus may influence energy balance, thereby linking IR to altered glucose metabolism, which could trigger migraine episodes [11]. Furthermore, metabolic changes associated with IR could disrupt the brain’s energy supply, potentially acting as a compensatory mechanism during migraine attacks [18]. Future research should include longitudinal studies to monitor changes in TyG index scores and IR markers over time in patients with migraines, providing insights into disease progression and causality. Additionally, efforts should focus on exploring the biochemical pathways underlying this relationship to uncover potential therapeutic targets. Expanding studies to include diverse populations to enhance the generalizability of the findings. Finally, intervention trials aimed at modifying IR levels are recommended to evaluate their impact on migraine outcomes.

While this study provides valuable insight, several limitations must be acknowledged, which warrant further validation of our conclusions. First, the psychological characteristics of individuals and specific medication use, which could significantly influence the outcomes, were not comprehensively addressed and should be considered in future research. Second, this research was based on a small-sample, single-center, cross-sectional study focused on the Chinese population, which limits the generalizability of the findings. Larger, multi-center studies across diverse populations are necessary to confirm these results. Third, longitudinal cohort studies, particularly involving newly diagnosed migraine patients, should be conducted to observe changes in IR and headache characteristics over time and elucidate the causal relationship between IR and migraines. Additionally, the potential protective mechanisms of IR or type 2 diabetes against migraines remain unclear, necessitating further investigation into how IR and other metabolic features interact with migraine pathogenesis. Finally, we recommend examining other potential confounding variables, such as lifestyle, dietary habits and genetic factors, which may also affect the study’s outcomes.

## 5. Conclusions

In summary, by integrating the results of cohort and cross-sectional studies, this study is the first to explore and discover the correlation between the IR marker TyG index scores and migraine disability in Chinese adults. Higher TyG index scores, which reflect a higher IR degree, may serve as a protective factor against migraine disability, particularly in young patients. This study offers a novel perspective on the relationship between IR and migraines, enhancing our understanding of the role of IR or related glycemic features in the occurrence and progression of migraines. Further research may contribute to the identification of new therapeutic targets for migraine treatment.
